# Multipolar Plasmonic Resonances of Aluminum Nanoantenna Tuned by Graphene

**DOI:** 10.3390/nano11010185

**Published:** 2021-01-13

**Authors:** Zhendong Yan, Qi Zhu, Xue Lu, Wei Du, Xingting Pu, Taoping Hu, Lili Yu, Zhong Huang, Pinggen Cai, Chaojun Tang

**Affiliations:** 1 College of Science, Nanjing Forestry University, Nanjing 210037, China; zdyan@njfu.edu.cn (Z.Y.); nanlinzhuqi@njfu.edu.cn (Q.Z.); xlu@njfu.edu.cn (X.L.); puxingting@163.com (X.P.); fox_tphu@sina.com (T.H.); llyu@njfu.edu.cn (L.Y.); 2 College of Physics Science and Technology, Yangzhou University, Yangzhou 225002, China; wdu@yzu.edu.cn; 3 College of Physics and Electronic Engineering, Jiangsu Second Normal University, Nanjing 210013, China; huangzhong89@126.com; 4 College of Science, Zhejiang University of Technology, Hangzhou 310023, China; caippgg@zjut.edu.cn

**Keywords:** graphene, multiple Fano resonances, dark mode, metamaterial

## Abstract

We numerically investigate the multipolar plasmonic resonances of Aluminum nanoantenna tuned by a monolayer graphene from ultraviolet (UV) to visible regime. It is shown that the absorbance of the plasmonic odd modes (*l* = 1 and *l* = 3) of graphene–Al nanoribbon structure is enhanced while the absorption at the plasmonic even modes (*l* = 2) is suppressed, compared to the pure Al nanoribbon structure. With the presence of the monolayer graphene, a change in the resonance strength of the multipolar plasmonic modes results from the near field interactions of the monolayer graphene with the electric fields of the multipolar plasmonic resonances of the Al resonator. In particular, a clear absorption peak with a high quality (*Q*)-factor of 27 of the plasmonic third-order mode (*l* = 3) is realized in the graphene–Al nanoribbon structure. The sensitivity and figure of merit of the plasmonic third-order mode of the proposed Graphene–Al nanoribbon structure can reach 25 nm/RIU and 3, respectively, providing potential applications in optical refractive-index sensing.

## 1. Introduction

Aluminum plasmonics is a rapidly growing field of nanoscience due to interest in both its scientific research and its promising potential applications [1,2,3,4]. Localized surface plasmon resonances (LSPR) [5,6] of Al nanostructures in the ultraviolet (UV) and visible range have been reported in several geometries, including spheres [7,8], disks [9,10], rods [2,11], and triangles [12,13]. Due to a low-cost alternative to the traditional noble metals (gold and silver), applications of the Al plasmonic nanostructures include enhanced light–mater interactions [14], full color display [15], UV fluorescence [16], UV surface enhanced Raman spectroscopy [17], label-free biosensing [18,19] and light harvesting [20,21].

In recent years, the research on LSPRs of Al nanostructures has mainly been focused on the fundamental electric or magnetic dipole modes [2,7,8,9,22,23,24]. Nevertheless, these fundamental resonant modes of Al nanostructures with poor *Q*-factor suffer from both strong radiative and non-radiative losses in UV and visible range [25]. In contrast, multipolar plasmonic high-order resonance modes [2,26,27] of Al nanoantennas with high *Q*-factor due to minimal non-radiative losses have attracted great attention as optical ultrahigh-sensitive sensors [28], multipolar radiations of quantum emitters [29], exciton–plasmon coupling [30,31] and nanolasing [32,33,34]. The multipolar high-order plasmonic modes have been experimentally investigated by a powerful tool of electron energy loss spectroscopy [1,2,10], revealing the spatial and spectral distributions. However, there are seldom reports on optical excitations of multipolar high-order plasmonic modes owing to their low excitation efficiency in UV range [35], which hinders the practical applications. Therefore, finding a way to effectively tune multipolar high-order resonant modes of Al nanoantenna excited by light with strong absorbance and highQ-factor is a prerequisite for its widespread applications.

Graphene, a flat monolayer of carbon atoms arranged in a honeycomb lattice, has been drawing tremendous attention owing to its electronic and optical properties for graphene-based photonics and optoelectronics devices in recent years [36,37,38,39,40,41]. From mid-infrared to THz range, the monolayer graphene can support the dynamic control of its plasmonic modes by tuning Fermi levels, resulting in enhanced optical absorption of graphene-based metamaterials [36,37,38]. However, from UV to near-infrared range, a poor optical absorption with universal value of 2.3% [42] of a monolayer graphene at a normal angle of incidence limits the applications of graphene-based optoelectronics devices. It is due to graphene’s optical response being dominated by interband transitions with no plasmonic response, acting as a passive lossy conductive surface. Recently, various mechanisms have been presented to enhance the absorption of graphene-based metasurfaces from UV to near-infrared range [28,43,44] by the strong interaction of the monolayer graphene with resonant metasurfaces. Zhou el. al. [44] and Yan et al. [28] reported that graphene–dielectric/metal metamaterial could be designed to enhance the UV ultranarrow absorption of graphene by the optical resonance modes supported by the dielectric or metal metamaterial. On the other hand, it is also important to understand the monolayer graphene-mediated resonant metasurfaces since the passive response acts as a precursor for the calibration of the metamaterial and plasmonic device designs. Li et al. [45,46] demonstrated that the changes in the transmission amplitude of the Fano resonance mode and the dipolar mode was observed owing to the interactions between the monolayer graphene and asymmetric spit-ring resonators in the terahertz range. Such prospect of switch-off effect of the resonance in metasurfaces is quite in demand for the potential applications based on light–matter interaction. However, in the UV range, optical intensity of metallic multipolar high-order plasmonic resonances mediated by graphene has not yet been reported.

In this paper, we demonstrate the multipolar plasmonic resonances of Al nanoribbon structure tuned by utilizing a monolayer graphene in UV and visible regime. Compared with the pure Al nanoribbon (AlNR) structure, the absorbance and electric fields at the plasmonic odd modes (*l* = 1 and *l* = 3) of graphene-Al nanoribbon (G-AlNR) structure are both enhanced. On the contrary, the absorption and the electric fields at the plasmonic even resonant mode (*l* = 2) are simultaneously decreased. It results from the near field interactions of the monolayer graphene with the electric fields of the multipolar plasmonic resonances of the Al resonator. It is worth noting that a clear enhancement of the absorption peak with a high *Q*-factor of 27 of the plasmonic high-order mode with *l* = 3 is obtained in the G-AlNR structure. The sensitivity and figure of merit of the third-order mode of the proposed G-AlNR structure can reach 25 nm/RIU and 3, respectively, providing practical applications such as optical refractive-index sensing.

## 2. Methods

**Figure 1:**
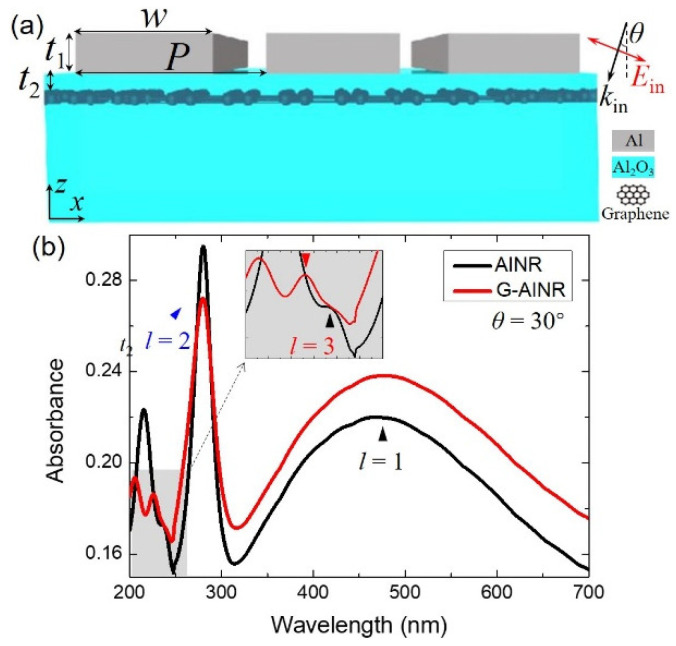
(**a**) Schematic of the proposed hybrid graphene–Al nanoribbon (G–AlNR) structure on Al_2_O_3_ substrate. The parameters *P*, *w*, *t*_1_ and *t*_2_ represent the period, width of the AlNR, thickness of the AlNR and the vertical distance between graphene and AlNR. The parameters *θ* represents the inclined incident angle. The length of AlNR is infinite along the *y* direction. (**b**) Absorbance of the G-AlNR and AlNR structures under TM light with inclined incident angle *θ* of 30°.

The schematic of the proposed hybrid graphene-Al nanoribbon (G-AlNR) structure on alumina (Al_2_O_3_) substrate is indicated in Figure 1a. The monolayer graphene is inserted into the Al_2_O_3_ substrate to avoid the contact and carrier transport between AlNR and graphene. The period, width of the AlNR, height of the AlNR and the vertical distance between graphene and AlNR are denoted as *P*, *w*, *t*_1_ and *t*_2_, respectively. The length of AlNR is infinite along the *y* direction. The monolayer graphene is considered as a passive and lossy thin film with a thickness (*t*_g_) of 0.34 nm. Unlike the previous study of graphene from infrared to terahertz, the surface conductivity (*σ*_g_) of graphene in the UV range can be described by a Fano model according to the many-body effects [43,47]:

(1) $$\sigma_{g}(\omega )=\frac{\sigma_{\mathrm{CB}}(\omega )\cdot {\left(q+E_{n}\right)}^{2}}{1+E_{n}{}^{2}}$$

(2) $$E_{n}=\frac{\hbar \omega -E_{r}}{\Gamma /2}$$

where *ћ*, *w* and *E*_n_ represent the Plank constant, the angular frequency of incident wave and the normalized energy by width Г = 0.78 eV relative to the resonance energy *E*_r_ = 5.02 eV of the perturbed exciton, respectively. *σ*_CB_(*w*) is the continuum background obtained by the calculation of a many-body system which denotes the response away from the singularity. The Fano parameter *q* determines the asymmetry of the conductivity line shape, which describes the excitonic transition strength to the unperturbed band transitions. Here, *q* is fixed at −1. Then, the permittivity of graphene is expressed as $\varepsilon_{g}=1+i\sigma_{g}/\left(\varepsilon_{0}\omega t_{g}\right)$. *ω* and *ε*_0_ are the angular frequency of incident wave and vacuum permittivity. The refractive index of Al_2_O_3_ the substrate is set as 1.76. The dielectric permittivity of Al is taken from the literature [48]. The optical performance of the proposed G-AlNR structure was investigated by the commercial software package “EastFDTD, version 5.0” based on the well-known finite difference time domain method. Due to the length of AlNR along the *y* direction being infinite, the simulations of optical properties of GNRs were performed in a 2D module. Periodic boundary conditions are used along the *x*-axis, and two perfectly matched layers (PML) are set along the *z* direction to eliminate boundary scattering of the electromagnetic waves. A Gauss pulse is set as light source, and the transmission spectra is obtained by Fourier transform. The electric field distributions on a plane can be recorded directly. In the regions of graphene and AlNR, the minimum mesh size is set to be 0.05 and 5 nm. For the other region, the homogeneous mesh size is set to be Δs = 20 nm, and the corresponding time step Δt = Δs/2*c*, where *c* is light speed in vacuum. The proposed hybrid structure is able to be fabricated experimentally through the following fabrication processes: a large-area graphene monolayer was firstly transferred to the top surface of the Al_2_O_3_ substrate. Then, an Al_2_O_3_ spacer with thickness of several nanometers is deposited on the top surface of the graphene monolayer by the electronic beam evaporation method. Finally, through electron beam lithography method, the periodical arrays of AlNR are fabricated directly on top of the Al_2_O_3_ spacer with high quality.

## 3. Results and Discussion

Figure 1b shows the calculated absorption spectra of the proposed G-AlNR structure (red solid line) and AlNR structure (black solid line) under the transverse magnetic (TM) light with inclined incident angle (*θ*) of 30°. The period (*P*), width (*w*), thickness (*t*_1_) of the AlNR and the vertical distance (*t*_2_) between graphene and AlNR are set as 110, 90, 10 and 1 nm, respectively. For graphene embedded in the Al_2_O_3_ substrate, there is a low absorption peak with a broad band centered at 265 nm, as shown in Figure S1, which is the optical dissipation mode of graphene [28]. For both the AlNR structure and G-AlNR structure, several absorption peaks are achieved, which range from 200 to 700 nm. These absorption peaks represent the multipolar plasmonic resonance modes of AlNR. The peaks depicted by *l* = 1, 2 and 3 denote the plasmonic fundamental mode (electric dipole mode), electric quadrupole mode and the third-order plasmonic mode of the AlNR structure. Such similar multipolar plasmonic resonance modes of Al nanoantennas have been experimentally investigated using electron energy loss spectroscopy [26]. The plasmonic fundamental mode (*l* = 1) and the third-order plasmonic mode (*l* = 3) are also called the plasmonic odd modes of AlNR that could be directly excited by incident light under normal incidence. On the contrary, the plasmonic even modes of AlNR such as the electric quadrupole mode (*l* = 2) could not be excited by incident light under normal incidence due to its net electric dipole moment being zero in the quasistatic limit. However, under inclined incident light with *θ* of 30°, all these plasmonic odd modes and even modes are excited simultaneously. Compared to AlNR, the corresponding absorption peaks (*l* = 1 and *l* = 2) of G-AlNR are located at the same position, while the position of corresponding absorption peak with *l* = 3 of G-AlNR slightly blueshifts, as shown in the inset of Figure 1b. It is shown that the absorbance at the plasmonic odd modes (*l* = 1 and *l* = 3) of the G-AlNR structure are larger than those of AlNR structure. In particular, the third-order plasmonic mode (*l* = 3) of AlNR is nothing more than a shoulder in the spectrum shown in the inset of Figure 1b, which is due to the low optical excitation efficiency for the metallic high-order plasmonic modes. Compared to AlNR, although only a small absorption peak is obtained under TM light with inclined incident angle (*θ*) of 30°, the *l* = 3 mode of G-AlNR with a narrow bandwidth is clearly enhanced. The full-width at half maximum (*FWHM*) and the corresponding *Q*-factor of the *l* = 3 mode of G–AlNR are extracted to be 8.5 nm and 27, respectively. The *Q*-factor of the *l* = 3 mode of G–AlNR is much larger than the *Q*-factor (*Q* = 2) supported by the plasmonic fundamental mode (*l* = 1) of the G–AlNR structure. The resonance strength of the *l* = 3 mode tuned by the inclined incident angle will be further discussed below. On the other hand, the absorbance at the plasmonic even mode (*l* = 2) of the G-AlNR structure is smaller than that of AlNR structure.

**Figure 2:**
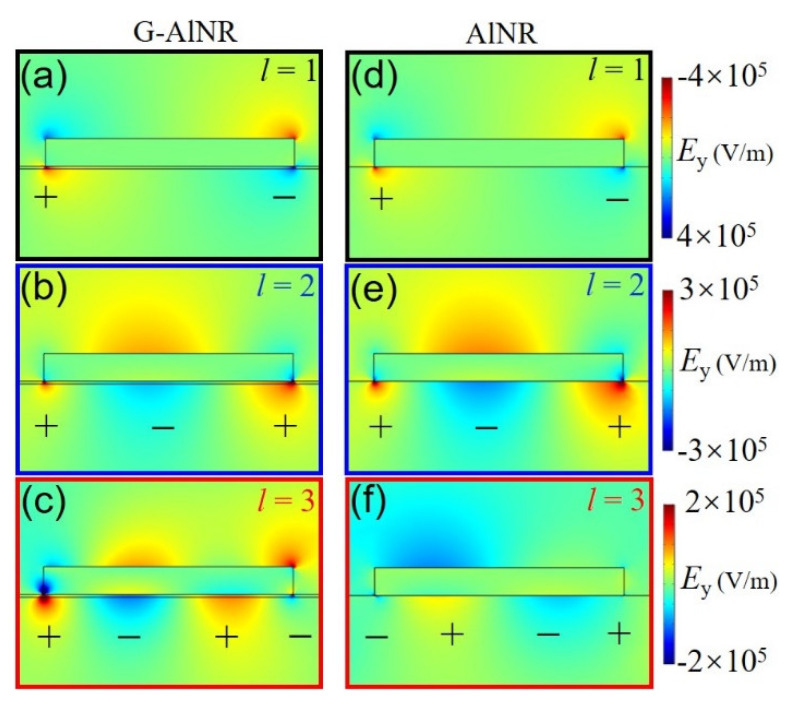
Electric field distributions of *E*_y_ and surface charge distribution from front view (in *xz* plane) of each plasmon mode for G-AlNR and AlNR: (**a**) *l* = 1, G-AlNR; (**b**) *l* = 2, G-AlNR; (**c**) *l* = 3, G-AlNR; (**d**) *l* = 1, AlNR; (**e**) *l* = 2, AlNR; and (**f**) *l* = 3, AlNR. Subplots (**a**,**d**), (**b**,**e**) and (**c**,**f**) are in the same scale, respectively.

The absorption of multipolar plasmonic resonances of AlNR tuned by a monolayer graphene can be explained by analyzing the electric field distributions. Figure 2 shows the electric field distributions of *E*_y_ and surface charge distribution of each plasmon mode for G-AlNR and AlNR. For *l* = 1, the electric field of the electric dipole mode of G-AlNR are slightly stronger than that of AlNR as shown in Figure 2a,d. For *l* = 2, the electric field of the electric quadrupole mode of G-AlNR are apparently smaller than that of AlNR, as shown in Figure 2b,e. For *l* = 3, the electric field of the plasmonic third-order mode of G–AlNR are apparently stronger than that of AlNR, as shown in Figure 2c,f. Therefore, the localized electric field and the absorbance of the plasmonic odd modes of AlNR are simultaneously enhanced, while the localized electric field and the absorbance of the plasmonic even mode of AlNR are simultaneously decreased by placing monolayer graphene in the dielectric substrate. Therefore, with the presence of the monolayer graphene below the AlNR, a change in the resonance strength of the multipolar plasmonic modes results from the near field interactions of the monolayer graphene with the electric fields of the multipolar plasmonic resonances of the AlNR. It is worth noting that the tunability of localized electric field of the plasmonic high-order mode (*l* = 2 and *l* = 3) at UV range coupled by graphene is larger than that of the plasmonic fundamental mode (*l* = 1) at visible range, owing to the optical broadband dissipation mode of graphene also centered at UV range.

**Figure 3:**
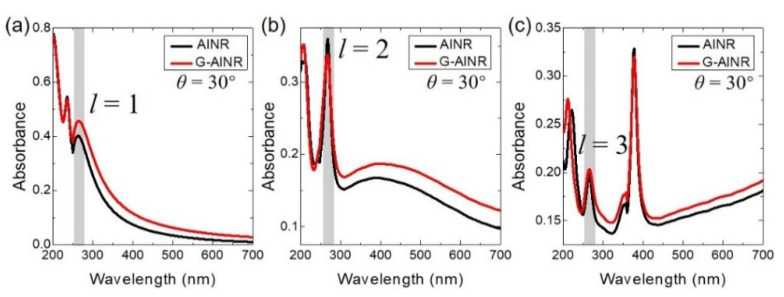
Absorbance of the G-AlNR and AlNR structures under TM light with incident angle (*θ*) of 30° with the three plasmon modes: (**a**) *l* = 1; (**b**) *l* = 2; and (**c**) *l* = 3. The resonant positions of the three plasmon modes of both the G-AlNR and AlNR structures are fixed at around 265 nm (gray zone) by choosing the right structural parameters (see text).

To comprehend the tunability of the absorption spectra of multipolar plasmonic resonances of AlNR tuned by a monolayer graphene, we simulated the absorption spectra of G-AlNR and AlNR under TM light with incident angle (*θ*) of 30° when the positions of the three plasmonic resonance modes (*l* = 1, 2 and 3) are fixed at 265 nm shown in the gray zone by choosing the proper structural parameters. In Figure 3a, the period (*P*), width (*w*), thickness (*t*_1_) of the AlNR and the vertical distance (*t*_2_) between graphene and AlNR are 110, 43, 20 and 1 nm, respectively. In Figure 3b, *P*, *w*, *t*_1_ and *t*_2_ are 110, 90, 15 and 1 nm, respectively. In Figure 3c, *P*, *w*, *t*_1_ and *t*_2_ are 160, 150, 15 and 1 nm, respectively. Figure 3a,c shows that the absorbance of the plasmonic odd modes (*l* = 1 and *l* = 3) at 265 nm of the G–AlNR structure are larger than those of AlNR structure. Figure 3b shows that the absorbance at the plasmonic even mode (*l* = 2) at 265 nm of the G-AlNR structure is smaller than that of AlNR structure, which exhibits the same variation tendency shown in Figure 1b.

**Figure 4:**
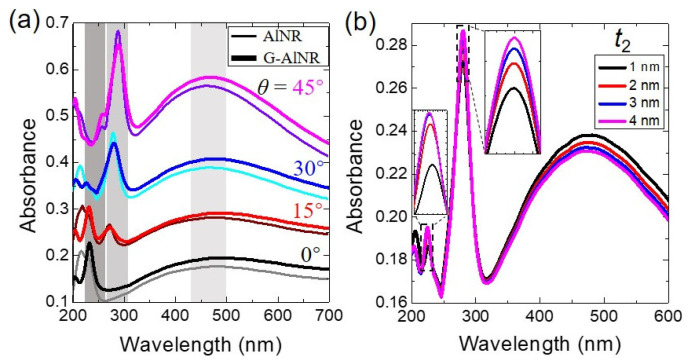
(**a**) The absorption spectra of the G-AlNR and AlNR structures under TM light with different incident angle (*θ*) from 0° to 45° with a step of 15°. (**b**) The absorption spectra of the G-AlNR with various the vertical distance (*t*_2_) between graphene and AlNR. The other parameters are the same as shown in Figure 1b.

We also analyzed the influence of the incident angle (*θ*) and the vertical distance (*t*_2_) between graphene and AlNR on the absorption of the G-AlNR structure under TM light. Figure 4a presents a series of absorption spectra of the G-AlNR (thick lines) and AlNR (thin lines) structures, with the incident angle (*θ*) increasing from 0° to 45° with a step of 15°. The absorption of multipolar plasmonic resonances (*l* = 1, 2 and 3) of AlNR tuned by a monolayer graphene under different *θ* shows the same variation tendency as that under *θ* = 30° shown in Figure 1b. In particular, it is found that the narrow absorption peak of the *l* = 3 mode is significantly enhanced when the inclined incident angle *θ* is decreased form 45° to 0°, which paves the way to practical applications. Figure 4b shows that the absorbance of the plasmonic fundamental mode (*l* = 1) of G-AlNR is slightly decreased while the absorbance of the plasmonic quadrupole mode (*l* = 2) and the plasmonic third-order mode (*l* = 3) are slightly increased as *t*_2_ is increased from 1 to 4 nm. It is worth noting that there is no plasmon hybridization between the AlNR and the bottom monolayer graphene to bring the resonant positions of the multipolar plasmonic modes of G-ALNR changed, which is due to the monolayer graphene acting as a passive and lossy conductive surface with no plasmonic response from UV to visible range. Such behavior in our proposed G-AlNR structure is different from the plasmonic coupling in the conventional metallic plasmonic materials such as Ag and Al in the UV range.

**Figure 5:**
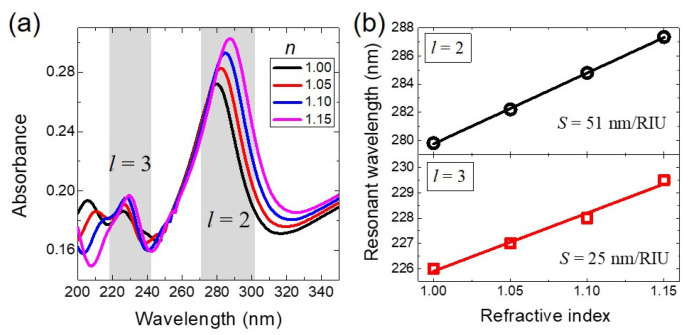
(**a**) Absorption spectra of the G–AlNR structure under TM polarization with inclined incidence angle (*θ*) of 30°, for different surrounding media with a refractive index changed from 1.00 to 1.15 with a step of 0.05. (**b**) Spectral shifts of the resonant positions of the plasmonic quadrupole mode (*l* = 2) and the third-order plasmonic mode (*l* = 3) with the surrounding refractive index changing from 1.000 to 1.040 with a step of 0.05. The geometrical parameters are the same as shown in Figure 1b.

Finally, we explore the potential of the plasmonic high-order mode of the G-AlNR structure for UV sensing applications. In Figure 5a, the absorption spectra of the G-AlNR structure embedded in different surrounding media are calculated under TM polarization with inclined incidence angle (*θ*) of 30°. The geometrical parameters are the same as shown in Figure 1b. The spectral redshifts of two peaks (*l* = 2 and *l* = 3) are observed when the refractive index of the surrounding media is changed from 1.00 to 1.15. The linearly fitted sensitivity *S* (*S* = Δλ/Δn) at UV range for the two peaks (*l* = 2 and *l* = 3) are 51 and 25 nm/RIU, respectively, as shown in Figure 5b. Here, Δ*λ* and Δ*n* are the wavelength shift and refractive index change, respectively. We further quantify the figure of merit (*FOM*), which is defined as the sensitivity divided by *FWHM* [30,31,32,33]. For the two peaks of *l* = 2 and *l* = 3, the two corresponding *FWHMs* are 27.5 and 8.5 nm. Thus, the *FOMs* of the two plasmonic modes (*l* = 2 and *l* = 3) of our proposed G-AlNR in UV range are 1.9 and 3, respectively, providing the practical applications such as ultraviolet refractive-index sensing.

## 4. Conclusions

In summary, we theoretically demonstrate the tunability of multipolar plasmonic resonances of Al nanoribbon structure in ultraviolet and visible regime controlled by utilizing monolayer graphene. Compared with the AlNR structure, the absorbance at the plasmonic odd modes (*l* = 1 and *l* = 3) of G–AlNR structure is enhanced while the absorption at the plasmonic even resonant mode (*l* = 2) of G–AlNR structure is decreased due to the interactions of the monolayer graphene with the electric fields of the multipolar plasmonic resonances of the Al nanoribbon. The electric fields of the multipolar plasmonic resonances of the Al nanoribbon shows the same variation tendency. In particular, the plasmonic high-order mode (*l* = 3) of G–AlNR with the full-width at half maximum of 8.5 nm and a high *Q*-factor of 27 is significantly enhanced. The sensitivity and figure of merit of the plasmonic high-order mode (*l* = 3) of the proposed G–AlNR structure can reach 25 nm/RIU and 3, respectively, providing practical applications such as the ultraviolet refractive-index sensing.

## Data Availability

Data available in a publicly accessible repository.

## Supplementary Materials

The following are available online at https://www.mdpi.com/2079-4991/11/1/185/s1, Figure S1: The Absorption spectrum of graphene embedded in the Al_2_O_3_ substrate.
