# Supplementary material for: Multipolar Plasmonic Resonances of Aluminum Nanoantenna Tuned by Graphene
Source: Nanomaterials (Basel). 2021 Jan 13;11(1):185. doi: 10.3390/nano11010185 (PMC7828546; doi:10.3390/nano11010185)
Supplement: Supplementary file 1 [file nanomaterials-11-00185-s001.pdf]

Supplementary Material

Multipolar Plasmonic Resonances of Aluminum Nanoantenna Tuned by Graphene

Zhendong Yan [1], Qi Zhu [1], Xue Lu [1], Wei Du [2], Xingting Pu [1], Taoping Hu [1], Lili Yu [1], Zhong Huang [3], Pinggen Cai [4] and Chaojun Tang [4,*]

[1] College of Science, Nanjing Forestry University, Nanjing 210037, China; zdyan@njfu.edu.cn (Z.Y.); nanlinzhuqi@njfu.edu.cn (Q.Z.); xlu@njfu.edu.cn (X.L.); puxingting@163.com (X. P.); fox_tphu@sina.com (T.H.); llyu@njfu.edu.cn (L.Y.)

[2] College of Physics Science and Technology, Yangzhou University, Yangzhou 225002, China; wdu@yzu.edu.cn

[3] College of Physics and Electronic Engineering, Jiangsu Second Normal University, Nanjing 210013, China; huangzhong89@126.com

[4] College of Science, Zhejiang University of Technology, Hangzhou 310023, China; caippgg@zjut.edu.cn

* Correspondence: chaojuntang@zjut.edu.cn

Contents

1. Absorption spectrum of graphene embedded in the Al2O3 substrate

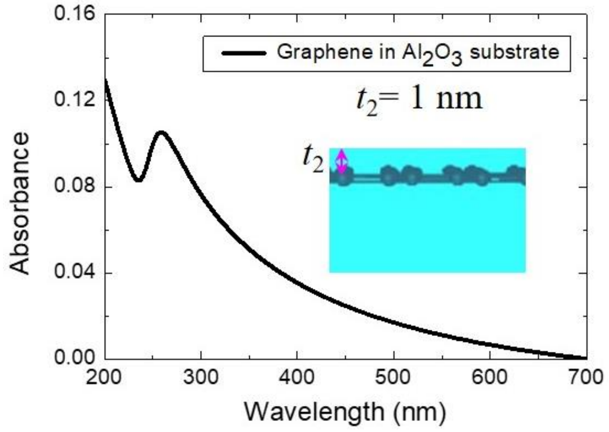

Figure S1. The Absorption spectrum of graphene embedded in the Al_2O_3 substrate. Graphene is inserted into the Al_2O_3 substrate with 1 nm below the upper surface.
